# A practical new strategy to prevent bile duct injury during laparoscopic cholecystectomy. A single-center experience with 5539 cases^1^

**DOI:** 10.1590/s0102-865020200060000007

**Published:** 2020-07-08

**Authors:** Peizhong Shang, Bing Liu, Xiaowu Li, Jianjun Miao, Ruichang Lv, Weilin Guo

**Affiliations:** I MD, Department of General Surgery, the Hospital of PLA 81st Group Army, Zhangjiakou, Hebei, China. Conception of the study, technical procedures, final approval.; II MD, Department of General Surgery, the Hospital of PLA 81st Group Army, Zhangjiakou, Hebei, China. Analysis of data, manuscript writing.; III MD, Department of General Surgery, the Hospital of PLA 81st Group Army, Zhangjiakou, Hebei, China. Technical procedures.; IV MD, Department of General Surgery, the Hospital of PLA 81st Group Army, Zhangjiakou, Hebei, China. Acquisition and analysis of data.

**Keywords:** Cholecystectomy, Laparoscopic, Gallbladder, Cholecystitis, Bile Ducts, Anatomy

## Abstract

**Purpose:**

Bile duct injury (BDI) is a catastrophic complication of cholecystectomy, and misidentification of the cystic anatomy is considered to be the main cause. Although several techniques have been developed to prevent BDI, such as the “critical view of safety”, the infundibular technique, the rates remain higher during laparoscopic cholecystectomy (LC) than during open surgery. We, here, propose a practical new strategy for ductal identification, that can help to prevent laparoscopic bile duct injury.

**Methods:**

A retrospective study of 5539 patients who underwent LC from March 2007 to February 2019 at a single institution was conducted. The gallbladder infundibulum was classified by its position located on an imaginary clock with the gallbladder neck as the center point of the dial, 3-o’clock position as cranial, 6-o’clock as dorsal, 9-o’clock as caudal, and 12-o’clock as ventral, as well as the axial position. Patient demographics, pathologic variables and infundibulum classification were evaluated. Detailed analysis of ductal identification based on gallbladder infundibulum position was performed in this study. All infundibulum positions were recorded by intraoperative laparoscopic video or photographic images.

**Results:**

All the patients successfully underwent LC during the study period. No conversion or serious complications such as biliary injury occurred. Gallbladders with infundibulum of 3-o’clock position, 6-o’clock position, 9-o’clock position, 12-o’clock position, axial position were 12.3%, 23.4%, 28.0%, 4.2%, and 32.1%, respectively. The 3-o’clock and 12-o’clock position were pitfalls that might cause biliary injury.

**Conclusion:**

The gallbladder infundibulum as a navigator is useful for ductal identification to reduce BDI and improve the safety of LC.

## Introduction

Laparoscopic cholecystectomy (LC) has been regarded as the gold standard for patients with benign gallbladder diseases since its introduction in the late 1980’s and is one of the most widely performed abdominal surgical operations. Laparoscopic procedures have multiple advantages including less postoperative pain, smaller scars, shorter hospitalization, and an earlier return to full activity over open cholecystectomy^1^. However, the incidence for bile duct injury (BDI) remains more frequent than that seen in the era of open surgery^2^, where the BDI rates were only 0.1%-0.2%^3^.

Bile duct injury is a catastrophic complication of cholecystectomy. Not only can BDI cause disturbances in quality and quantity of life^4,5^, but it can result in litigation^6^. Much has been learned about the mechanism of BDI, for which misperception of normal anatomy is a most common reason, especially misidentification of the common bile duct as the cystic duct^7-9^. Therefore, it is crucial to find reliable anatomical landmarks and obtain a satisfactory exposure for ductal identification during LC.

Several methods have been advocated for target identification in LC. The “critical view of safety” (CVS) proposed by Strasberg is the most widely accepted technique on avoidance of biliary injury^10,11^. However, it is thought that BDIs may occur while the surgeon is attempting to achieve the CVS^12^. Another commonly used method is the infundibular technique, which is easier and takes less dissection than CVS^11^. Regrettably, this method appears to be unreliable and misleading in certain circumstances, where the cystic duct is hidden^13^. A straightforward and reliable method of ductal identification is warranted for LC.

In our experience, the error trap of the infundibular technique can be avoided by recognizing the variations in the anatomy of the gallbladder infundibulum. We found a regular relationship among the infundibulum, the cystic duct, the common hepatic duct, and the common bile duct. The proper identification of this relationship is critical to avoid pitfalls.

This study presents a series of 5539 cases, who underwent LC at a single center with no biliary injury, and describe in detail how to avoid biliary injury in LC with the gallbladder infundibulum as a navigator for ductal identification. We believe that this practical new strategy can help to reduce the BDI and improve the safety of LC.

## Methods

A series of patients with benign gallbladder diseases who underwent LC at our center from March 2007 to February 2019 were retrospectively reviewed. Patients with the following conditions were excluded: acute or atrophic cholecystitis with severe inﬂammation and ﬁbrosis of the hapatocystic triangle, Mirizzi syndrome, and malignancies. This study was approved by the Research Ethics Committee of the Hospital of PLA 81^st^ Group Army, Hebei and written informed consents were obtained from all the participants. All preoperative diagnoses were made by ultrasonography and magnetic resonance cholangiopancreatography (MRCP). Demographic and pathologic information of included patients was recorded (Table 1).

**Table 1:** Patient demographic and pathologic variables.

Characteristic	
Age, y, mean (range)	56.2 (22-84)
Female, n (%)	4193 (75.7)
Symptomatic, n (%)	4858 (87.7)
Pathology, n (%)	
Chronic cholecystitis	3811(68.8)
Acute cholecystitis	615 (11.1)
Subacute cholecystitis	598 (10.8)
Atrophic cholecystitis	166 (3.0)
Gallbladder polyps	415 (7.5)

### *Classification of the gallbladder infundibulum*

**Figure 1:**
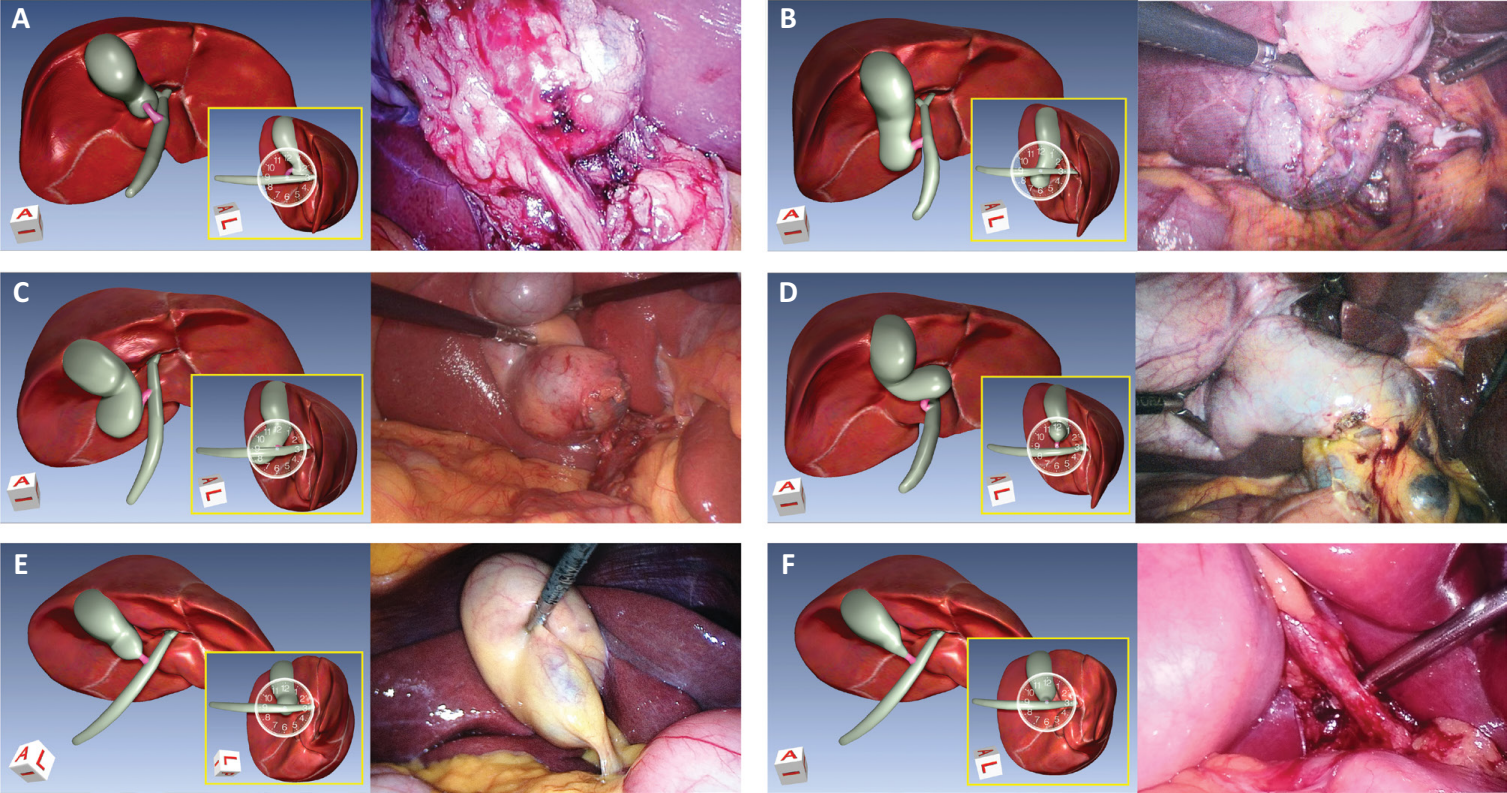
Illustrations of gallbladder infundibulum with A. 3-o’clock position, B. 6-o’clock position, C. 9-o’clock position, D. 12-o’clock position, E. Axial position type I position, and F. Axial type II position.

The gallbladder infundibulum is variable in size and shape, and when this funnel-shaped portion is eccentrically inflated, it is called a Hartmann’s pouch^14^. According to our experience, this pouch lies at different directions with respect to the cystic duct and the common bile duct with a certain regularity. As the patient is placed in a supine position, and the surgeon stands on the left side of the patient with a line-of-sight from left to right, an imaginary clock facing the surgeon is vertically placed with the gallbladder neck as the center point of the dial. The Hartmann’s pouch is assigned a location on this imaginary clock face to distinguish its positions (with the 3-o’clock position as cranial, 6-o’clock as dorsal, 9-o’clock as caudal, and 12-o’clock as ventral) (Fig. 1 A-D). In addition, if the infundibulum portion is uniformly dilated without a Hartmann’s pouch, we refer to this condition as axial position. Furthermore, if the infundibulum with axial position is obviously inflated, it is defined as type I (Fig. 1E), otherwise, it is defined as type II (Fig. 1F).

### *Surgical technique*

All procedures were performed under general anesthesia. The patient was placed in supine position, then in reverse Trendelenburg position with left side down after insufflation of the abdomen. The surgeon stands on the patient’s left, while the assistant is on the right side. The camera operator stands to the surgeon’s left. A three-port technique was routinely used (10mm periumbilical port, 10mm subxiphoid port, and 5mm lateral subcostal port). For some difficult gallbladders or a requirement to explore the common bile duct, one more port was needed (5mm medial subcostal port).

The abdomen was entered via a 10-mm umbilical incision using a closed (Veress needle) technique, and a 30° laparoscope was introduced through the umbilical trocar. Then the surgeon’s 10-mm trocar and the assistant’s 5-mm trocar were typically inserted under direct vision. The surgeon pushed the liver and the gallbladder cranially and the assistant pushed the gastric antrum and duodenum caudally to reveal the infundibulum, then the first photograph was taken. The first and key step was to identify the position of infundibulum, which can help to find the cystic duct. The assistant should grasp the gallbladder body or infundibulum with a toothed grasper and retract in different direction to expose the gallbladder neck and cystic duct. In the scenario of infundibular calculus, the surgeon should first dislodge and “milk” the stone back up into the gallbladder body. For 3-o’clock position, Hartmann’s pouch protrudes into the hepatocystic triangle, the infundibulum should be retracted rightwards, ventrally, and caudally to reveal the gallbladder neck and cystic duct. For 6-o’clock position, the cystic duct is usually easy to find when the infundibulum is retracted rightwards, ventrally, and caudally. For 9-o’clock position, the cystic duct arises from cranial aspect of the infundibulum and is usually covered when the infundibulum achieves considerable size. The assistant should retract rightwards, ventrally, and cranially to reveal the gallbladder neck and cystic duct. For 12-o’clock position, the infundibulum always overlies the common bile duct. The surgeon should strip the peritoneum and retract rightwards and ventrally to expose the cystic duct. For axial position, the cystic duct arises from the apex of the gallbladder, which is easy to be identified. After identification and dissection of the cystic duct, the second photograph was taken. Finally, the cystic duct was incised and the assistant grasped the most dilated portion of the infundibulum to re-identify the clipped cystic duct, then the third photograph was taken. The gallbladder was then dissected from the liver bed with hook cautery and ﬁnally removed via the 10mm subxiphoid port. If possible, the procedures were also recorded by laparoscopic camera. All the infundibulum positions were recorded in the operation notes.

## Results

During the study period, 5539 patients underwent a successful laparoscopic operation, consisting of 4193 females and 1346 males (mean age 56.2 years, range 22–84 years). Most (68.8%) of the patients underwent surgery for chronic cholecystitis, followed by 11.1% for acute cholecystitis, 10.8% for subacute cholecystitis, and 3.0% for atrophic cholecystitis, while the remaining 7.5 % for gallbladder polyps.

The mean postoperative hospital stay was 3.92 days (range 1–9 days). 4559 cases (82.3%) were operated with a three-port technique and 980 (17.7%) cases required an additional port. No conversion to an open procedure or complications such as bleeding, infection occurred. There was no incidence of bile duct injury during surgery or the follow-up period. The classification of the gallbladder infundibulum was recorded by photographs, and 615 cases were also recorded by intraoperative videos, of which the 3-o’clock position, 6-o’clock position, 9-o’clock position, 12-o’clock position, axial position type I, and axial type II was 12.3%, 23.4%, 28.0%, 4.2%, 24.3%, and 7.8%, respectively (Table 2).

**Table 2:** Classification of the gallbladder infundibulum.

position	3-o’clock	6-o’clock	9-o’clock	12-o’clock	axial type I	axial type II
n	681	1296	1551	233	1346	432
%	12.3	23.4	28.0	4.2	24.3	7.8

## Discussion

There has been a sharp rise in the incidence of BDI since the introduction of LC. Although the surgical technique and laparoscopic equipment, as well as the surgeon’s learning curve have improved, the incidence of BDI is still higher in comparison to the era of open surgery, with a rate noted from approximately 0.2% to about 1.1%, and remains one of the most serious iatrogenic surgical complications^2,6,8,12,15^. Despite the scarcity of clinical evidence on its precise mechanism, clinical experience shows that most BDI occurs as a result of misidentification of the cystic structures, such as misidentification of the common bile duct, an aberrant bile duct or the common hepatic duct as the cystic duct^7-9^. Therefore, a reliable method of ductal identification is crucial to prevent BDI.

There have been several methods advocated for ductal identification during LC, such as the “critical view of safety” (CVS) technique, the infundibular technique, and fundus-down cholecystectomy^11^. The CVS technique has been used worldwide, and is adopted as the gold standard or a mandatory step for assessment of biliary anatomy during LC by some centers^7^. This method requires complete cleaning of all fatty and fibrous tissue in the hepatocystic triangle and dissection of the lower portion of the gallbladder off the liver bed to ensure only two structures (the cystic duct and artery) entering the gallbladder^10,16^. However, it seems that the CVS is an ideal destination for target identification but how to get there is not yet described in detail, and biliary injury may occur on the way to get there^12,17^. The infundibular method, which necessitates a view of a funnel-shaped structure resembling the junction of gallbladder and cystic duct, despite taking less dissection and easier than CVS, has been proved unreliable in the presence of sever inflammation^13,18^. The fundus-down cholecystectomy is an alternative in difficult circumstance; however, extreme vasculobiliary injuries tend to occur when this method is performed^19^.

During our early experience with performance of over one thousand LCs, we found a regular relationship between the gallbladder infundibulum, the surrounding biliary structures, and the infundibulum as a navigator was extremely helpful for ductal identification ^20^. In this series, we further evaluated the incidence and morphology of the infundibulum, and established a new standard method of performing LC based on this feature.

In general, the gallbladder is divided into four parts: fundus, body, infundibulum, and neck^21^. Hartmann’s pouch, which is defined as the eccentric prominent of the infundibulum, is one of the most important structures in the era of LC^22,23^. However, there are different opinions on the incidence and nature of this pouch^14,24^. Consistent with previous research, we found Hartmann’s pouch a frequent but inconstant feature of not only pathologic but also physiologic gallbladders. It should be noted that there was a higher prevalence (68.5%) of Hartmann’s pouch observed in this study than some previous studies, which was about 52%^14^ or only 4%^24^, respectively. For the rest of the gallbladders with no Hartmann’s pouch in this series, the infundibulum was further divided into two types, those with centric bulging, and those without, which was defined as axial position type I, and axial position type II, respectively.

All infundibulum positions in this study were recorded by intraoperative photographic images, part of which were also recorded by laparoscopic videos. Stefanidis and colleagues^25^ analyzed ten LC videos; the results show that only two of the ten surgeons obtain adequate CVS before division of the cystic duct. Sanford and colleagues^26^ proposed a simple method of photographing both anterior and posterior views of CVS during LC; the results show that intraoperative doublet photography can record CVS accurately and increase the safety of LC. In this study, photographs were taken at the time when the infundibulum was revealed, after dissection and clipping of the cystic duct, and after division of the cystic duct. Thus, at least three photographs were taken, to make sure that the infundibulum position was recorded accurately.

Although our method for target identification is based on the gallbladder infundibulum, it is worth pointing out that there is an essential difference between our method and the infundibulum technique. The anatomic rationale for cystic identification of infundibulum technique is based on three-dimensional demonstration of a flaring tunnel shape as the cystic duct-gallbladder junction^13,18^, which might be deceptive when the cystic duct was hidden. In our method, the first and the key step is to identify the position of infundibulum, especially the 3-o’clock and the 12-o’clock, rather than the structure appearing to flare. Once entry into the abdomen, the infundibulum should be revealed, followed by identification of the position. If the 6-o’clock or 9-o’clock position is not observed, the surgeon must be aware of the possibility of the 3-o’clock or the 12-o’clock position. For the 3-o’clock position, Hartmann’s pouch protrudes into the hepatocystic triangle, even adjacent to the porta hepatis. Dissection should be very careful in case that the common hepatic duct, right hepatic duct, right hepatic artery, or gallbladder artery will be injured^27^. For the 12-o’clock position, the cystic duct is hidden by Hartmann’s pouch overlying the common bile duct, making the surgeon misidentifying the common bile duct as the cystic duct, then the common bile duct will be transected, which is one of the most serious injuries^13^. It is important to note that the position is not a precise point but within a certain range. For example, Hartmann’s pouch located at the 2-o’clock to 4-o’clock is considered as the 3-o’clock, and so on.

Our method can be used in combination with other techniques to minimize the risk of biliary injury, especially in patients with acute cholecystitis with nondissectable scarring or severe ﬁbrosis of the Calot’s triangle. For those LCs with adhesions and fibrosis surrounding the GB, it is difficult to identify the local anatomy or the position of infundibulum, as well as achieving a CVS, a bail-out procedure should be chosen, such as subtotal cholecystectomy, fundus-down technique, and open conversion^8,28-30^. In our experience, the separation can be started at the fundus in difficult LCs to expose infundibulum, then to identify the cystic duct in light of the position of infundibulum^28^. In the cases with frozen triangle of Calot, subtotal fenestrating cholecystectomy is an alternative, and the cystic duct orifice can be identified from the interior of the gallbladder by infundibulum position, then be closed from the inside^8,29^. Furthermore, some other factors are also important for performing a safe LC, such as understanding the potential for aberrant anatomy, adequate exposure, recognizing when bail-out procedures are needed, as described by the SAGES Safe Cholecystectomy Task Force^2,7^.

This study is limited in single-center data. As the low event rate of BDI, the use and evaluation in future multicenter studies with large numbers of patients of our method as a valuable strategy for ductal identification is therefore required.

## Conclusions

The gallbladder infundibulum is variable in shape but has a certain regularity, which is useful as a navigator for ductal identification. This, therefore, can help to reduce BDI and improve the safety of LC.
